# Flavonoids Restore Platinum Drug Sensitivity to Ovarian Carcinoma Cells in a Phospho-ERK1/2-Dependent Fashion

**DOI:** 10.3390/ijms21186533

**Published:** 2020-09-07

**Authors:** Yifat Koren Carmi, Hatem Mahmoud, Hazem Khamaisi, Rina Adawi, Jacob Gopas, Jamal Mahajna

**Affiliations:** 1 Department of Nutrition and Natural Products, Migal-Galilee Research Institute, Kiryat Shmona 11016, Israel; ifat.koren@gmail.com (Y.K.C.); hatem.gu13@gmail.com (H.M.); hazemkh@migal.org.il (H.K.); rinaadawi@gmail.com (R.A.); 2 Shraga Segal Department of Microbiology, Immunology and Genetics, Ben Gurion University of the Negev, Beer Sheva 8410501, Israel; jacob@bgu.ac.il; 3 Department of Oncology, Soroka University Medical Center, Beer Sheva 8410501, Israel; 4 Department of Biotechnology, Tel-Hai College, Kiryat Shmona 11016, Israel; 5 Department of Nutritional Sciences, Tel-Hai College, Kiryat Shmona 11016, Israel

**Keywords:** ovarian cancer, chemoresistance, flavonoid, ERK

## Abstract

Ovarian cancer (OC) is the second most common type of gynecological malignancy; it has poor survival rates and is frequently (>75%) diagnosed at an advanced stage. Platinum-based chemotherapy, with, e.g., carboplatin, is the standard of care for OC, but toxicity and acquired resistance to therapy have proven challenging. Despite advances in OC diagnosis and treatment, approximately 85% of patients will experience relapse, mainly due to chemoresistance. The latter is attributed to alterations in the cancer cells and is also mediated by tumor microenvironment (TME). Recently, we reported the synthesis of a platinum (IV) prodrug that exhibits equal potency toward platinum-sensitive and resistant OC cell lines. Here, we investigated the effect of TME on platinum sensitivity. Co-culture of OC cells with murine or human mesenchymal stem cells (MS-5 and HS-5, respectively) rendered them resistant to chemotherapeutic agents, including platinum, paclitaxel and colchicine. Platinum resistance was also conferred by co-culture with differentiated murine adipocyte progenitor cells. Exposure of OC cells to chemotherapeutic agents resulted in activation of phospho-ERK1/2. Co-culture with MS-5, which conferred drug resistance, was accompanied by blockage of phospho-ERK1/2 activation. The flavonoids fisetin and quercetin were active in restoring ERK phosphorylation, as well as sensitivity to platinum compounds. Exposure of OC cells to cobimetinib—a MEK1 inhibitor that also inhibits extracellular signal-regulated kinase (ERK) phosphorylation—which resulted in reduced sensitivity to the platinum compound. This suggests that ERK activity is involved in mediating the function of flavonoids in restoring platinum sensitivity to OC co-cultured with cellular components of the TME. Our data show the potential of combining flavonoids with standard therapy to restore drug sensitivity to OC cells and overcome TME-mediated platinum drug resistance.

## 1. Introduction

Each year, about 240,000 women worldwide are diagnosed with ovarian cancer (OC). The disease is treated with surgery and chemotherapy but unfortunately, patients will frequently relapse, even after an initial positive response. Development of resistance to chemotherapy and the associated development of malignant abdominal fluid (ascites) is a major challenge for patients with advanced OC.

OC ranks as the second most common type of gynecological malignancy and has poor survival rates [1]. Platinum-based chemotherapy represents the standard of care for OC, but toxicity and acquired resistance have proven challenging [2,3]. The apoptosis-promoting activity of chemotherapeutic agents is mediated by a variety of signaling pathways [4] and consequently, alterations in specific signaling pathways might result in chemoresistance. Moreover, chemoresistance in OC is also mediated by tumor microenvironment (TME). An increasing number of studies are demonstrating the importance of TME in tumor progression, metastasis and chemoresistance. The TME consists of soluble factors and non-cancerous cells, including cancer-associated fibroblasts, mesenchymal stem cells (MSCs), macrophages, and other peritoneal cells, such as adipocytes and mesothelial cells [5].

OC has a clear predilection for metastasis to the omentum, an organ primarily composed of adipocytes. It is unclear why OC cells preferentially home to and proliferate in the omentum. Recent findings have revealed that primary human omental adipocytes promote homing, migration, and invasion of OC cells. Moreover, co-culture of adipocytes and OC cells led to the direct transfer of lipids from the former to the latter and promoted tumor growth in vitro and in vivo, as well as resistance to chemotherapy and radiotherapy [6]. Interestingly, free fatty acid oxidation rate in OC cells increased in co-culture with adipocytes [7]. Furthermore, a recent study has shown that preadipocytes and adipocyte-derived factors reduce the sensitivity of Her2+ breast tumor cells to trastuzumab [8].

More than one-third of OC patients present with malignant ascites at initial diagnosis. The presence of ascites is a fundamental part of the recurrent disease [9,10]. The onset and progression of ascites is associated with poor prognosis and a deterioration in patients’ quality of life [9]. Malignant ascites acts as a reservoir for a complex mixture of soluble factors and cellular components, providing a proinflammatory and tumor-promoting microenvironment for the OC cells that could be associated with chemoresistance [11,12,13,14,15]. Ascites-derived malignant cells and the ascites microenvironment represent a major source of morbidity and mortality for OC patients. A number of signaling pathways have been suggested to be responsible for the TME-mediated drug resistance, including stromal cell-derived factor-1 (SDF-1 or CXCL12) and its receptors, CXCR4 and CXCR7 [16,17,18]. The Notch-signaling pathway has also been implicated in recurrent and chemoresistant OC [19,20,21,22]. Moreover, transcriptional upregulation of Mcl-1, a pro-survival Bcl-2 protein, by OC ascites constitutes an additional chemoresistance mechanism that is mediated by the extracellular signal-regulated kinase (ERK) 1/2-signaling pathway [23].

The ERK-signaling pathway plays a crucial role in almost all cell functions, including mediating a variety of antiproliferative events, such as apoptosis, autophagy, and senescence, in vitro and in vivo [24]. ERK activity can promote either intrinsic or extrinsic apoptotic pathways by inducing mitochondrial cytochrome c release or caspase 8 activation, permanent cell-cycle arrest or autophagic vacuolization [24]. Previous reports have shown that DNA-damaging agents such as platinum activate ERK1/2 kinase, which is directly involved in mediating stabilization of Bim, a BH3-only proapoptotic protein, and consequently, induce apoptosis in the treated cells [25,26].

Flavonoids are dietary polyphenols present in a wide variety of plants, fruit, vegetables, nuts, and teas [27]. More than 6000 flavonoids have been isolated and identified, and divided into subclasses, among them the flavones, flavonols, flavanols, isoflavones, and isoflavans [28,29]. Flavonoid consumption may contribute to preventing diseases, including cardiovascular and neurodegenerative diseases, and cancer [30,31]. There is growing evidence of the anticancer activity of a variety of flavonoids [32,33,34,35]. Moreover, previous reports have shown flavonoid’s ability to affect the activity of specific signaling pathways, including ERK signaling, through either stimulation [36,37,38,39] or ERK1/2-signaling inhibition [40,41], accompanied by reactive oxygen species (ROS) generation [42].

In this study, we show that co-culture of OC cells with murine MSCs (MS-5) confers platinum drug resistance, accompanied by blockage of ERK 1/2 phosphorylation. The flavonoids fisetin and quercetin were active in restoring ERK phosphorylation, as well as sensitivity to platinum compounds. Cobimetinib, which inhibits ERK phosphorylation, resulted in reduced sensitivity to the platinum compound, consistent with the role of activated ERK1/2 in mediating platinum-induced apoptosis. Our data show the potential of combining flavonoids with standard therapy to restore drug sensitivity to OC cells and overcome TME-mediated platinum drug resistance.

## 2. Materials and Methods

### 2.1. Cell Lines

Human platinum-sensitive and resistant OC cell lines A2780 and A2780CisR, respectively (obtained from the American Type Culture Collection [ATCC], Manassas, VA, USA), were cultured in RPMI 1640 complete medium supplemented with 10% (*w*/*v*) fetal bovine serum (Biological Industries, Beit HaEmek, Israel), 1% (*w*/*v*) l-glutamine, 100 units/mL penicillin, and 0.1 mg/mL streptomycin. The human and murine MSC lines HS-5 and MS-5, respectively, and the murine adipocyte cell line 3T3-L1 (also obtained from ATCC) were maintained under the same conditions. All cell lines were grown at 37 °C in a humidified atmosphere with 5% CO_2_.

### 2.2. In-Vitro Experiments

Human OC cell lines A2780 and A2780CisR were plated at 8 × 10^3^ cells/cm^2^ in 25 cm^2^ or 75 cm^2^ cell-culture flasks and incubated in culture medium. They were incubated as a monoculture or as a co-culture with MS-5, HS-5 or 3T3-L1 cell lines, which were seeded at 4.27 × 10^3^ cells/cm^2^. After 24 h, cells were treated with platinum chemotherapy, consisting of 5 μM of our synthesized platinum (IV) prodrug (RJY13) or 25 μM cisplatin, or anti-microtubule chemotherapy, consisting of colchicine or paclitaxel, with or without inhibitory agents (50 μg/mL Oriental mistletoe extract, 10 μM quercetin,10 μM fisetin,10 μM myricetin or 2 μM cobimetinib). After additional 24 h incubation, the attached cells were collected using trypsin in RPMI medium. Cells were counted (the sample was diluted 1:1 in Trypan blue and cells were counted using a hemocytometer), then centrifuged at 3000 rpm (1000× *g*) for 5 min and washed with cold phosphate buffered saline (PBS) twice to obtain cell pellets.

### 2.3. Differentiation of 3T3-L1 Cells

Differentiation of 3T3-L1 cells to adipocytes was performed by plating 3 × 10^3^ cells/cm^2^ in DMEM, 10% fetal bovine serum (FCS), 100 units/mL penicillin, 0.1 mg/mL streptomycin, and 2 mM l-glutamine. After 24 h, the medium was replaced with medium containing 1 mM dexamethasone, 5 mg/mL insulin and 0.5 M 3-isobutyl-1-methylxanthine. After 48 h, the medium was replaced with DMEM containing 5 mg/mL insulin. This last step was repeated again 48 h later. Cells were fixed and tested for differentiation using Oil red O solution (Merck, Darmstadt, Germany). Oil red was extracted with isopropanol and absorbance at 492 nm was measured by spectrophotometer (Tecan, Männedorf, Switzerland).

### 2.4. Trypan Blue Exclusion Assay

Cells (2 × 10^5^/well) were plated in six-well plates. After 24 h, cells were treated with the specified agents. Solvent-treated samples were incubated with 1% (*w*/*v*) dimethyl sulfoxide. Cells were collected 72 h later, stained with 0.4% (*w*/*v*) Trypan blue solution (1:1, *v*/*v*), and counted using a hemocytometer [43].

### 2.5. Construction of A2780/CFP-YFP

A2780 cells were stably transfected with pECFP-DEVDR-Venus (Addgene plasmid #24537; http://n2t.net/addgene:24537; RRID: Addgene_24537) which was a gift from Peter Sorger. Transfection of the plasmid, which carries resistance to geneticin (G418) for selection, was performed with Xtreme Gene HP DNA Transfection Reagent (Sigma, Neustadt, Germany) according to the manufacturer’s instructions. G418-resistant fluorescent cells were selected and named A2780 Venus.

### 2.6. Immunoblotting

Protein analysis was performed by western blot protocol on an 8–12% acrylamide gel. Cell lysate samples were prepared for loading by adding lysis buffer (#9803 Cell Signaling Technology, Danvers, MA, USA) containing protease inhibitors (P8340 and P5726, Sigma, Neustadt, Germany) and phosphatase inhibitor (P-1517, AG Scientific, San Diego, CA, USA) to the cell pellets. After 30 min, samples were centrifuged and supernatants were tested for protein concentration using DC™ Protein Assay (Bio Rad, Hercules, CA, USA) and determining absorbance at 630 nm. Samples were lysed in lysis buffer and 50–60 μg protein per monoculture sample, or 100–120 μg protein per co-culture sample was loaded on the gel. Proteins were immunoblotted onto a nitrocellulose membrane (Schleicher and Schuell BioScience GmbH, Dassel, Germany) which was then blocked with 5% skim milk TBS/T, and incubated with the following antibodies: anti-cleaved poly (ADP-ribose) polymerase (PARP) (Asp214) (D64E10, Cell Signaling Technology), anti-α-tubulin (sc-8035, Santa Cruz, TX, USA), anti-GFP (#2956, Cell Signaling Technology) and anti-phospho-ERK1/2 (Thr202/Tyr204) (D13.14.4E, Cell Signaling Technology) according to the manufacturers’ instructions. Secondary antibodies, HRP-linked anti-rabbit (#7074, Cell Signaling Technology) and anti-mouse (NB7539 Novus, Centennial, CO, USA) were used according to the manufacturers’ instructions. Chemiluminescence was performed with SuperSignal™ West Pico PLUS Chemiluminescent Substrate (Thermo Fisher Scientific, Waltham, MA, USA) and imaged using an HP imager. Densitometry was performed with ImageQuant v8.2 software.

### 2.7. PathScan^®^ Cleaved PARP Assay

Cleaved PARP in cell lysates was measured using PathScan^®^ Cleaved PARP (Asp214) Sandwich ELISA Kit (Cell Signaling Technology) according to manufacturer instructions. This ELISA kit has the ability to detect cPARP from human and murine origin.

## 3. Results

### 3.1. Co-culture with MSCs Confers Drug Resistance to OC Cells

We followed the drug susceptibility of OC cells growing in co-culture with murine and human MSCs (MS-5 and HS-5, respectively), and with murine adipocyte progenitor (3T3-L1), monitored their sensitivity to platinum compounds, and ran a PARP-cleavage assay to determine apoptosis induction.

**Figure 1:**
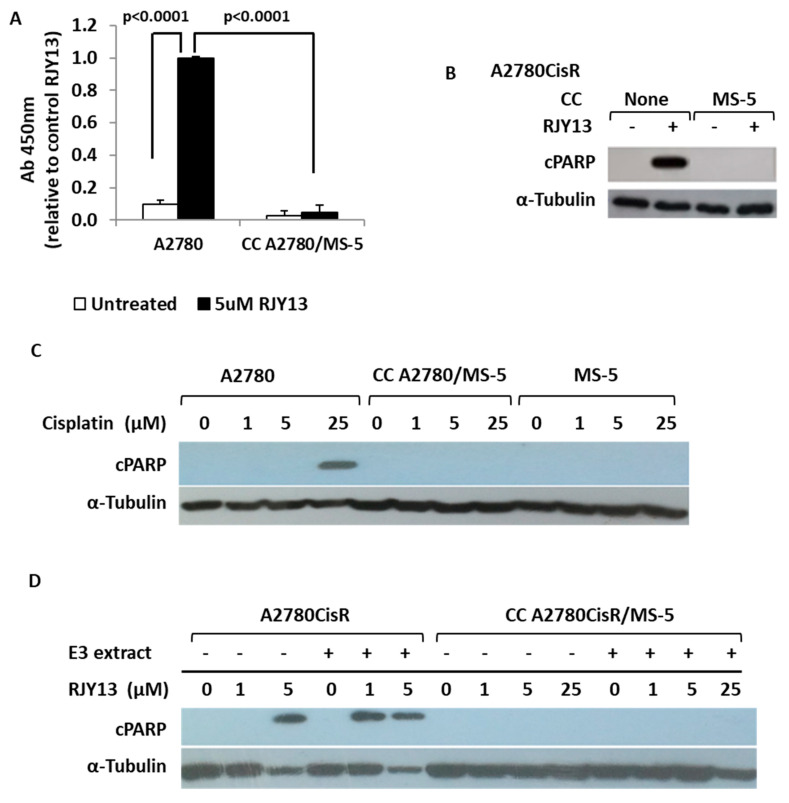
MS-5 confers drug resistance to ovarian cancer (OC) cells. (**A**) OC cisplatin-sensitive cells (A2780) were exposed to 5 μM of RJY13 in the presence and absence of MS-5 and levels of cleaved polymerase (PARP) were monitored by PARP ELISA kit. (**B**) OC cisplatin resistant cells (A2780CisR) were exposed to 5 μM of RJY13 in the presence and absence of MS-5 and levels of cleaved PARP were monitored by immunoblotting. (**C**) OC cisplatin sensitive cells (A2780) were exposed to different concentrations (1–25 μM) of cisplatin in the presence and absence of MS-5 and levels of cleaved PARP were monitored by immunoblotting. (**D**) OC cisplatin resistant cells (A2780CisR) were exposed to 1 or 5 μM of RJY13 in the presence and absence of MS-5 and levels of cleaved PARP were monitored by immunoblotting. A2780cisR co-cultured with MS-5 were also exposed to 1, 5, and 25 μM of RJY13 in the presence and absence of oriental mistletoe extract (E3 extract) at 50 μg/mL (**D**). α-Tubulin was used as a loading control. CC, co-culture.

Initially, we used an ELISA kit to monitor the ability of our synthesized platinum prodrug RJY13 [44] to induce apoptosis by monitoring cleavage of nuclear PARP in OC cells exposed to RJY13, as a marker of cells undergoing apoptosis [45]. Upon exposure to RJY13, significant induction of cleaved PARP was observed in OC cells, whereas only marginal PARP cleavage was observed in OC cells co-cultured with MS-5 cells and exposed to RJY13 (Figure 1A). Next, we monitored PARP cleavage by immunoblotting in a co-culture experiment using a species-selective PARP antibody that recognizes the cleaved forms of PARP from human origin, but not from murine origin (Figure 1B). Exposure of OC cells to cisplatin (Figure 1C) or RJY13 (Figure 1B,D) resulted in significant induction of apoptosis in A2780 cells (Figure 1C), as well as in A2780CisR cells (Figure 1B,D); however, they failed to induce apoptosis in A2780 or A2780CisR cells co-cultured with MS-5 cells (Figure 1). Moreover, the presence of Oriental mistletoe extract, which enhances RJY13 activity against cisplatin-resistant OC cells (A2780CisR) [46], failed to induce apoptosis of the same cells when they were co-cultured with MS-5 cells (Figure 1D).

### 3.2. Human MSCs Confer Platinum Drug Resistance to OC Cells

**Figure 2:**
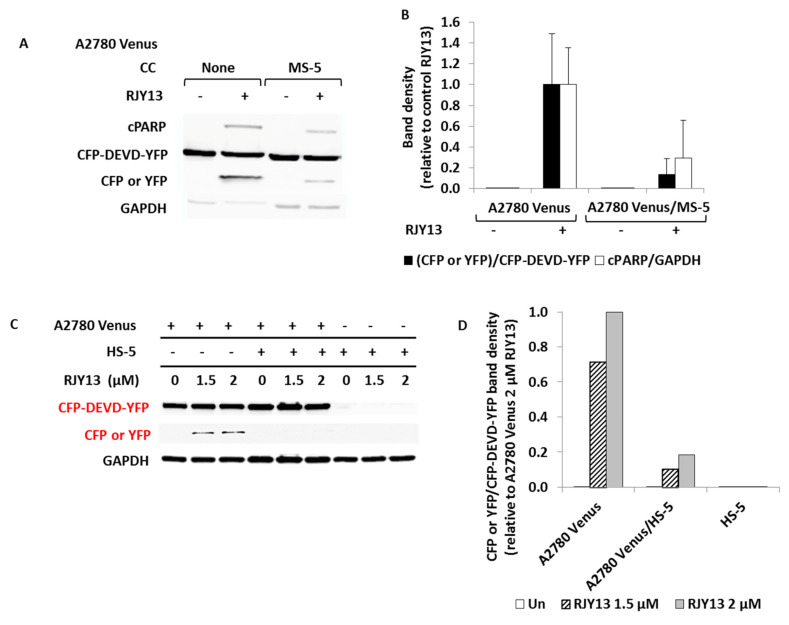
HS-5 cells confer drug resistance to OC cells. OC cisplatin sensitive cells carrying pECFP-DEVDR-Venus plasmid (A2780 Venus) cells were co-cultured with MS-5 (**A**,**B**) or HS-5 (**C**,**D**) and exposed to 5 μM RJY13 or 1.5 or 2 μM RJY13, respectively. Levels of cleaved CFP/YFP (CFP or YFP, **A**,**C**) and cleaved human PARP (cPARP, **A**) were determined by immunoblotting (**A**,**C**). Relative cPARP levels as well relative levels of cleaved CFP were calculated and plotted (**B**,**D**). GAPDH was used as a loading control. CC, co-culture.

To explore the ability of HS-5 cells to affect platinum sensitivity of OC cells, we modified our previous approach of using the human-selective cleaved PARP antibody, because it cannot distinguish cleaved PARP originating from OC cells from that originating from HS-5 cells. We therefore used A2780 Venus cells, which stably express the fluorescent reporter protein EC-RP (a fusion protein) consisting of CFP (cyan fluorescent protein) fused to YFP (Venus yellow fluorescent protein). The two fluorescent proteins are connected by a flexible linker that contains the caspase 3-cleavable sequence DEVDR [47]. Upon exposure of A2780 Venus cells to platinum compounds that lead to apoptosis induction, caspase 3 is activated, causing cleavage of the DEVDR linker that connects the two fluorescent proteins and producing cleaved CFP/YFP protein. The modified assay was validated using A2780 Venus cells co-cultured with MS-5 cells, and monitoring cleavage of PARP and CFP/YFP following exposure to RJY13. Figure 2A shows that cleavage of CFP/YFP behaved comparably to that of PARP. Next, we monitored the ability of HS-5 cells to confer chemoresistance in OC cells. We noticed that HS-5 cells were 2- to 3-fold more sensitive to RJY13 than MS-5 cells (data not shown). Figure 2B shows that A2780 Venus cells expressed the large CFP/YFP fusion protein, arguing that no apoptosis was taking place. However, upon exposure to RJY13, cleaved CFP/YFP (29 kDa) was observed (Figure 2B). In co-culture with HS-5 cells, however, no cleaved CFP/YFP was observed, arguing that the presence of HS-5 cells reduces platinum sensitivity of OC cells, similar to the data obtained using MS-5 cells (Figure 1).

### 3.3. Co-Culture with Differentiated Murine Adipocyte Progenitor (3T3-L1) Cells Confers Drug Resistance to OC Cells

**Figure 3:**
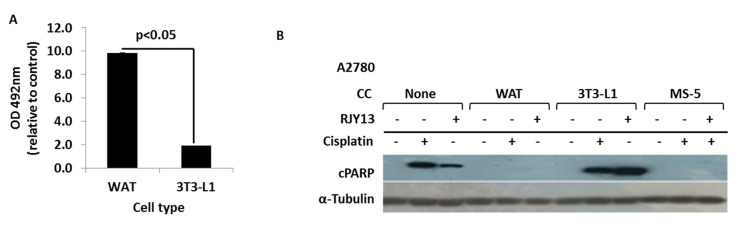
Differentiated 3T3-L1 cells confer drug resistance to OC cells. Murine adipocyte progenitor 3T3-L1 cells were stimulated to differentiate to white adipocytes (WAT) and the presence of fat droplets was determined using Oil red staining (**A**). A2780 cells exposed to platinum compounds (5 μM RJY13 or 25 μM cisplatin) were co-cultured with differentiated (WAT) and undifferentiated 3T3-L1 cells as well as MS-5 cells (**B**). Levels of cleaved PARP (cPARP) were monitored in the different samples by immunoblotting using human selective anti-cPARP antibody (**B**). α-Tubulin was used as a loading control. The experiment was repeated twice with comparable outcomes. CC, co-culture.

We explored the possibility of adipocyte cells affecting platinum drug sensitivity of OC cells, utilizing 3T3-L1 cells that were stimulated to differentiate to white adipocytes (WAT) based on a well-established protocol [48]. Differentiated and undifferentiated adipocyte cells were co-cultured with OC cells and exposed to RJY13, and PARP cleavage was monitored. Figure 3 shows that differentiated 3T3-L1 cells (WAT) stimulated the accumulation of fat droplets, as measured by Oil red assay (Figure 3A). We then monitored PARP cleavage by immunoblot (Figure 3B), and found that platinum treatment (RJY13 or cisplatin) stimulated PARP cleavage, and that the presence of MS-5 cells conferred drug resistance, as previously demonstrated (Figure 1). Interestingly, no PARP cleavage was observed in samples of OC cells co-cultured with differentiated 3T3-L1 cells. In contrast, co-culture of undifferentiated (progenitor) 3T3-L1 cells with OC cells showed minor effects on the amount of cleaved PARP after exposure to RJY13. This argues that differentiated 3T3-L1 cells confer drug resistance to platinum compounds, whereas undifferentiated, progenitor 3T3-L1 cells do not.

### 3.4. Co-Culture with MS-5 Cells Blocks Platinum-Induced Phosphorylation of ERK1/2 in OC Cells

Previous reports have shown that platinum exposure induces the activation of ERK1/2 kinase, which is directly involved in mediating apoptosis in the treated cells [25,26].

**Figure 4:**
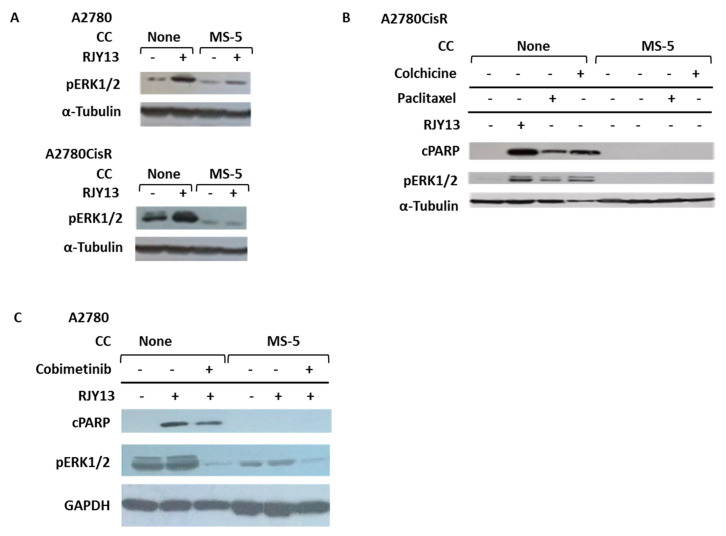
Co-culture of MS-5 cells with OC cells blocks ERK1/2 activation. OC cells sensitive (A2780; **A**) and resistant (A2780CisR; **A**,**B**) to cisplatin were treated with 5 μM RJY13 (**A**,**B**), 50 nM colchicine, or 25 nM paclitaxel (B) alone or in co-culture with MS-5 cells. Levels of cleaved PARP (cPARP) and phospho-ERK1/2 (pERK1/2) were monitored. A2780 cells were treated with RJY13 (5 μM) in mono or co-culture with MS-5 cells in the presence or absence of 2 μM cobimetinib (**C**). α-Tubulin (**A**,**B**) and GADPH (**C**) were used as loading controls. CC, co-culture.

Figure 4A shows that treatment of OC cells with RJY13 resulted in upregulation of phospho-ERK1/2 in both OC cell lines (A2780 and A2780CisR). Interestingly, co-culture of OC cells with MS-5 cells resulted in blocked ERK1/2 activation (Figure 4A) following exposure to platinum compound, arguing that MS-5 co-culture interferes with platinum-mediated ERK1/2 activation. Similarly, exposure of OC cells to paclitaxel or colchicine resulted in induction of apoptosis in OC cells, as measured by PARP cleavage (Figure 4B), whereas co-culture of OC cells with MS-5 cells prevented PARP cleavage and abolished the sensitivity of OC cells to the different drugs. Exposure of OC cells to paclitaxel or colchicine also resulted in upregulation of phospho-ERK1/2, correspondingly, co-culture of OC cells with MS-5 cells resulted in blockage of ERK1/2 phosphorylation (Figure 4B). To further support the involvement of ERK in platinum-induced apoptosis, we used cobimetinib, an orally bioavailable small-molecule inhibitor of mitogen-activated protein kinase kinase 1 (MEK1) which also inhibits ERK phosphorylation [49], and monitored its ability to affect platinum-induced apoptosis of OC cells. Figure 4C shows that cobimetinib significantly inhibited ERK1/2 phosphorylation in A2780 cells and in parallel, moderately affected the ability of platinum to induce apoptosis, as evidenced by a moderate reduction in the levels of cleaved PARP. This argues that ERK1/2 activation mediates, in part, the apoptosis-inducing function of platinum compounds in A2780 cells.

### 3.5. Flavonoids Restore Platinum Drug Sensitivity to OC Cells Co-Cultured with MS-5 Cells

**Figure 5:**
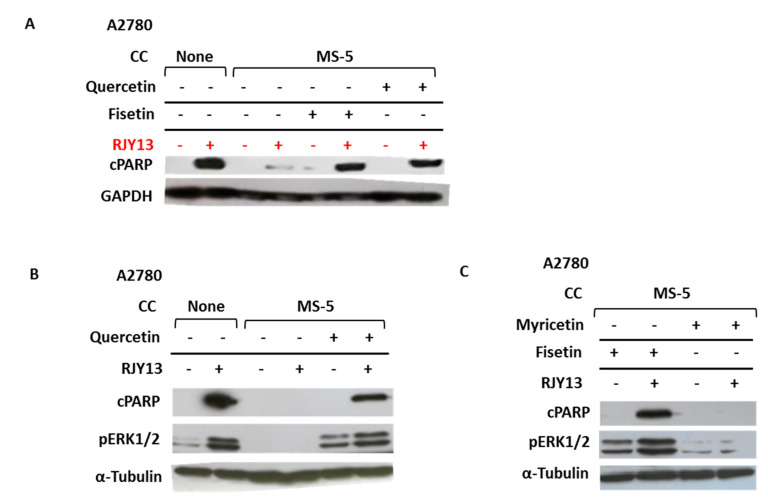
Fisetin and quercetin activate ERK1/2 in co-culture with MS-5 cells and restore OC cell platinum drug sensitivity. OC cells were treated with RJY13 in the presence and absence of MS-5 cells and levels of cleaved PARP (cPARP; **A**–**C**) and phospho-ERK1/2 (**B**,**C**) were monitored. Fisetin (**A**,**C**), quercetin (**A**,**B**), or myricetin (**C**) were added at 10 μM along with RJY13. α-Tubulin was used as a loading control. CC, co-culture.

To explore the role of ERK1/2 in MS-5-mediated drug resistance, we asked whether downregulation of phospho-ERK1/2 as a result of co-culture with MS-5 cells is responsible for mediating platinum drug resistance in OC cells. We tested the hypothesis that fisetin and related flavonoids might be useful for restoring platinum sensitivity of OC cells by activating the ERK1/2 pathway. Figure 5A shows that the presence of MS-5 cells prevented apoptosis induction of OC cells exposed to RJY13, as monitored by the presence of cleaved PARP. However, sensitivity of OC cells to RJY13 was restored when 10 μM of either quercetin or fisetin was added with RJY13, as evidenced by the presence of cleaved PARP protein in OC cells treated with RJY13 in co-culture with MS-5 cells (Figure 5A). Moreover, fisetin and quercetin were both active in stimulating ERK1/2 phosphorylation in the co-culture, in both the presence and absence of RJY13 (Figure 5B,C). Interestingly, the flavonoid myricetin, which exhibited no activity in restoring drug sensitivity to OC cells, also failed to significantly activate ERK1/2 (Figure 5C).

## 4. Discussion

Primary and acquired drug resistance are major challenges in cancer therapy, in particular when using platinum compounds [50]. The molecular mechanisms that underlie this chemoresistance are largely unknown. However, decreased platinum accumulation, elevated drug inactivation by metallothionein and glutathione, and enhanced DNA-repair activity have been suggested [51]. Moreover, acquired and inherited resistance of cancer cells has been reported to also be mediated by altered molecular mechanisms and activation of a variety of signaling pathways [52,53]. In addition to chemoresistance which is mediated by alterations within the cancer cells, an increasing number of reports also implicate the TME in tumor chemoresistance [5,54]. Moreover, in OC, the effect of TME is even more pronounced, especially in cases with malignant ascites [54]. Of special interest are the MSCs, which are multipotent progenitors that provide a significant advantage to tumor cells in terms of enhanced proliferation, metastasis and neovascularization [55,56,57,58,59]. In-vitro studies have shown that co-culture of OC cells with MSCs induces broad transcriptomic changes [60,61,62]. In this study, we explored the effect of the MSCs MS-5 and HS-5, and murine 3T3-L1 cells on drug susceptibility of OC cells. The cells were grown in co-culture and we monitored their sensitivity to platinum compounds using the PARP-cleavage assay for assessment of apoptosis induction [44]. Initially, exposure of OC cells (A2780 or A2780CisR) to the platinum compounds cisplatin or our novel bis-octanoatoplatinum (IV) complex RJY13 [44] resulted in apoptosis induction. However, when OC cells were co-cultured with MSCs (MS-5 or HS-5), a significant increase in resistance to the platinum drugs was observed.

This argued that the presence of MSCs confers drug resistance to OC cells. Interestingly, murine MS-5 and human HS-5 cells were equally active (Figure 1 and Figure 2) in conferring resistance to the platinum drug, implying that human OC cells also recognize and respond to the murine signals that might be mediated by soluble factors or by cell–cell interactions. Moreover, platinum drug resistance mediated by co-culture with MSCs was observed in both cisplatin-sensitive and cisplatin-resistant OC cells. Furthermore, drug resistance mediated by co-culture with MSCs was observed in both OC cell types when exposed to a variety of chemotherapeutic agents. In addition, the presence of Oriental mistletoe extract, which enhances RJY13 activity against cisplatin-resistant OC cells (A2780CisR) [46], failed to overcome MSC-mediated drug resistance in those same cells. Interestingly, co-culture with MS-5 cells has been shown to confer drug resistance to other cancer cells, including leukemia (CML cells) and breast cancer (MCF7) cells using Abl tyrosine kinase inhibitors or cisplatin, respectively (Mahajna et al., unpublished data).

A recent report illustrated the capacity of 3L3-L1 preadipocytes to protect human leukemia cell lines from chemotherapeutic agents, an effect which was independent of cell contact and associated with the increased expression of antiapoptotic factors Pim-2 and Bcl-2 [6]. Moreover, Bochet et al. [63] demonstrated that cancer-associated adipocytes can also promote radio-resistance and proposed that this effect could be due to IL-6 secretion and phosphorylation of Chk1. In addition, adipocytes have been implicated in chemoresistance of cells from solid tumors, such as breast cancer cells exposed to doxorubicin [64]. We found that only differentiated adipocytes were active in conferring platinum drug resistance to OC cells; the undifferentiated 3T3-L1 cells were not. This indicates that some of the factors produced by differentiated adipocytes [65] are responsible for mediating drug resistance. The nature of these factors remains to be elucidated.

The drug resistance observed in our study was not limited to platinum compounds; other chemotherapeutics, such as paclitaxel and colchicine, failed to promote apoptosis in OC cells co-cultured with MSCs, arguing that this phenomenon is not cell-type specific or chemotherapeutic-specific, but is a general phenomenon (Figure 4). Our results (Figure 4) showed that treatment of OC cells with platinum, paclitaxel or colchicine causes upregulation of phosphorylated ERK1/2 levels. This observation is consistent with previous reports showing that the apoptosis-promoting activity of many therapeutic agents is mediated by activation of the ERK-signaling pathway [4]. However, co-culture with MSCs prevented this activation. The detailed mechanism responsible for this activity remains to be elucidated.

Consistent with our finding are the results reported by Villedieu et al. [66], showing that acquisition of cisplatin resistance in OAW42-R ovarian carcinoma cells is associated with loss of ERK activation in response to cisplatin. Moreover, the synthetic compound 2-[3-(2,3-dichlorophenoxy) propylamino] ethanol was active in inducing ERK activation and consequently restored cisplatin sensitivity in OC cells.

Similar to previous reports [36,37,38,39], the flavonoids fisetin and quercetin were active in stimulating ERK1/2 phosphorylation in OC cells. In contrast, others have claimed that the apoptosis-inducing function of fisetin and quercetin is mediated through ERK1/2-signaling inhibition [40,41], accompanied by ROS generation [42]. The differences in the outcome of ERK activation are probably due to differences in cellular content. Our study revealed that myricetin, which did not restore drug sensitivity to OC cells, also failed to activate phospho-ERK1/2 (Figure 5C). This argues that not all flavonoids activate ERK and restore platinum drug sensitivity to OC cells. We are currently conducting a structure–activity relationship study to elucidate the nature of the functional groups in fisetin and quercetin (or other flavonoids) that are required for ERK stimulation and consequently, for resensitizing OC cells to platinum’s function.

Modulation of the ERK-signaling pathway has been implicated in mediating the activity of some chemotherapeutic agents, including DNA-damaging compounds such as platinum [25,26]. Interestingly, other reports have demonstrated that it is not activation, but inhibition of ERK signaling that is associated with the antitumor effect [67]. However, a growing number of studies have suggested that under certain conditions, aberrant ERK activation can promote cell death. The proapoptotic function of the ERK pathway is well documented for apoptosis induced by DNA-damaging agents, such as etoposide and platinum compounds [68,69]. It seems that sustained ERK activation is required for induction of cell death. DNA-damaging agents, such as cisplatin or etoposide, activate ERK and catalyze the formation of ROS, which in turn prolong ERK activation, and this might be the crucial mechanism for ERK-induced cell death [70]. Subcellular localization of ERK is tightly regulated, and the outcome of ERK-mediated cell death might also rely on aberrant subcellular localization. Indeed, apoptosis induced by estradiol [71], tamoxifen [72], and others is correlated with sustained nuclear ERK activity [73]. Interestingly, some compounds that induce nuclear ERK activity were also associated with the production of ROS [74,75] via inhibition of dual-specificity phosphatases, which regulate phosphorylation of ERK, p38, and others. Thus, sustained activation of ERK in different subcellular compartments is not tolerated and results in different forms of cell death. However, the molecular mechanism mediating MSC-mediated blockage of ERK phosphorylation, and how flavonoids such as fisetin and quercetin reactivate ERK activity, have yet to be explored.

It is currently unclear how co-culture with MSC blocks platinum-induced activation of ERK1/2. Possible candidates are the soluble factors secreted by MSCs. This possibility remains to be explored. Cell–cell interactions might also participate in blocking platinum-induced ERK activation. Cell–cell interactions have been implicated in mediating drug resistance that relies on the activity and presence of adhesion molecules such as CXCR4/CXCL12 [76,77,78,79], the JAK/STAT pathway [80], and others. CXCR4/CXCL12 signaling has been reported to mediate protective effects on OC cells against hyperthermia [61]. In a preliminary experiment, we showed that JAK/STAT and CXCR4/CXCL12 signaling might not be involved in mediating platinum chemoresistance to OC cells co-cultured with MSCs. This is because exposure to AMD3100/3465 (CXCR4 antagonist) or Ruxolitinib (JAK1/2 inhibitor) was ineffective at restoring platinum sensitivity to OC cells co-cultured with MSCs (results not shown), suggesting that other or multiple signaling pathways are involved in MSC mediation of chemoresistance to OC cells.

The present in-vitro study illustrated the involvement of MSCs as well as adipocytes in conferring chemoresistance in OC cells. However, in an in-vivo setting, we expect crosstalk and cooperation between the different components of the TME to alter platinum drug sensitivity. It is also possible that other metabolites or additional signaling pathways are involved in mediating OC chemoresistance. For example, metabolites such as fatty acids have been reported to confer drug resistance in OC cells [81]. Moreover, exosomes isolated from human umbilical cord (HuC) MSCs containing microRNAs modulating the PI3K/Akt-signaling pathway were implicated in conferring MSC-induced chemoresistance in OC [82]. Thus, it is reasonable to speculate that multiple signaling pathways, including ERK, PI3K/Akt and others, as well as other metabolites, are cooperating to promote OC chemoresistance [81,82].

In conclusion, our data show that platinum drug susceptibility in OC cells is affected by the presence of murine and human MSCs (MS-5 and HS-5, respectively) in co-culture. Platinum compounds failed to induce apoptosis in OC cells co-cultured with MSCs. Moreover, apoptosis of platinum-treated OC cells was correlated with stimulation of ERK1/2 phosphorylation, the latter being blocked in OC cells co-cultured with MSCs. The flavonoids fisetin and quercetin, which activated ERK1/2 phosphorylation in co-culture, were also active in restoring platinum sensitivity to OC cells. Our data argue that induction of platinum-mediated apoptosis is dependent on ERK1/2 activation, and that chemoresistance in co-culture is a result of blocked ERK1/2 activation. Restoration of ERK1/2 phosphorylation by flavonoids results in restored sensitivity to platinum drugs and abolishment of TME-mediated chemoresistance.
